# Diagnostic Value of ANA Titer, Anti-ds-DNA, Anti-nRNPSm, and Sm Antibodies in Early Atypical Systemic Lupus Erythematosus

**DOI:** 10.12669/pjms.42.9.14966

**Published:** 2026-09

**Authors:** Jianqiao Zhang, Meichen Liu, Qing Liu, Dong Chen, Meng Zhang

**Affiliations:** 1 Jianqiao ZhangDepartment of Laboratory, Affiliated Hospital of Hebei University, Baoding 071000, Hebei, China; 2 Meichen Liu Department of Laboratory, Affiliated Hospital of Hebei University, Baoding 071000, Hebei, China; 3 Qing Liu Department of Laboratory, Affiliated Hospital of Hebei University, Baoding 071000, Hebei, China; 4 Dong Chen Department of Laboratory, Hebei Provincial Mental Health Center (The Sixth People’s Hospital of Hebei Province), Baoding 071000, Hebei, China; 5 Meng ZhangDepartment of Laboratory, Affiliated Hospital of Hebei University, Baoding 071000, Hebei, China

**Keywords:** Atypical, Combined diagnosis, Diagnostic value, Early stage, Systemic lupus erythematosus

## Abstract

**Objective:**

To explore the diagnostic value of antinuclear antibody(ANA) titer, anti-double-stranded DNA(anti-ds-DNA) antibody, anti-nuclear ribonucleoprotein/Smith(anti-nRNPSm) antibody, and Smith (Sm) antibody for early atypical systemic lupus erythematosus(SLE).

**Methodology:**

This was a retrospective study. Forty-nine patients diagnosed with early atypical SLE admitted to Affiliated Hospital of Hebei University between January 2023 to June 2025 were assigned to Group-A, and 66 patients with other autoimmune diseases admitted during the same period were enrolled in Group-B. Compared the results of ANA, anti-ds-DNA, anti-nRNPSm and Sm antibody tests between the two groups, and analyzed the diagnostic value of the individual indicators and their combined detection for early atypical SLE.

**Results:**

There were statistically significant differences in the results of ANA, anti-ds-DNA, anti-nRNPSm and Sm antibodies between the two groups(P<0.05). Multivariate Logistic regression analysis confirmed that ANA, anti-ds-DNA, anti-nRNPSm and Sm were independent influencing factors for early atypical SLE (*P*<0.05). ROC curve analysis showed that the AUC of combined diagnosis was 0.856, with a predictive sensitivity of 87.8%, a specificity of 71.2%, and a predictive probability of 0.79. Significant differences in AUC were observed between ANA and anti-ds-DNA antibody, Sm antibody, or the four-antibody combination(*P*<0.05); between anti-ds-DNA antibody and the four-antibody combination(*P*<0.05); and between anti-nRNPSm antibody, Sm antibody and the four-antibody combination(*P*<0.05).

**Conclusion:**

ANA, anti-ds-DNA, anti-nRNPSm and Sm antibodies are major independent influencing factors for early atypical SLE. Combined detection of these indicators may effectively improve the diagnostic value for this disease.

## INTRODUCTION

Systemic lupus erythematosus(SLE) is characterized by complex and diverse clinical manifestations. Its early symptoms are often non-specific, making it prone to confusion with other rheumatic and immunological diseases, which may result in delayed diagnosis or misdiagnosis.^1^,^2^ Although the diagnostic value of biomarkers such as ANA, anti-ds-DNA, and anti-Sm antibodies for SLE has been well established, their diagnostic sensitivity and specificity are still relatively inadequate in patients with early atypical SLE.^3^,^4^ Most existing studies have focused on the validation of single antibodies or traditional diagnostic criteria. However, systematic analyses on the dynamic changes of antibody profiles and the efficacy of combined diagnosis in patients with early atypical SLE are relatively scarce. Therefore, exploring the value of combined detection of multiple antibodies for the diagnosis of early atypical SLE is an urgent need in current clinical research. Based on this, the authors conducted a retrospective analysis of clinical data from 49 patients with early atypical SLE and 66 patients with other autoimmune diseases admitted to our hospital. The aim of this study was to evaluate the diagnostic value of ANA, anti-ds-DNA, anti-nRNPSm, and Sm antibodies for early atypical SLE, thereby providing references for its early diagnosis, subsequent treatment, and rehabilitation.

## METHODOLOGY

This was a retrospective study. Forty-nine patients diagnosed with early atypical SLE admitted to Affiliated Hospital of Hebei University between January 2023 to June 2025 were assigned to Group-A, and 66 patients with other autoimmune diseases admitted during the same period were enrolled in Group-B. Patient data including demographic data, and baseline dosage were retrieved from electronic medical record systems. The sample size was calculated using the formula:


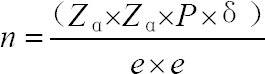


Where, ***n*** is the total sample size, ***α***=0.05 (*Z_α_*=1.96), ***P*** is the overall probability (0.667), **δ** is the probability difference (0.338), and **e** is the allowable error (0.086). The final calculated total sample size was 115 cases.

In Group-A, there were 24 males and 25 females, aged 24–53 years with a mean age of (41.6±5.8) years. In Group-B, there were 32 males and 34 females, aged 22–55 years with a mean age of (41.9±6.1) years. The types of autoimmune diseases in Group-B included 22 cases of connective tissue diseases, 18 cases of Sjögren’s syndrome, 20 cases of rheumatoid arthritis, and 6 cases of vasculitis. No statistically significant differences were observed in gender or age between the two groups (*P*>0.05), indicating good comparability.

### Ethical approval:

The study was approved by the Institutional Ethics Committee of Affiliated Hospital of Hebei University (No.:HDFYLL-KY-2022-020; approval Date: December 30, 2022) and written informed consent was obtained from all participants.

### Inclusion criteria:

- Age ≥18 years.
- Patients in Group-A were diagnosed with early atypical SLE, in accordance with the diagnostic criteria for atypical early SLE specified in the *Chinese Guidelines for the Diagnosis and Treatment of Systemic Lupus Erythematosus* formulated by the Rheumatology Branch of the Chinese Medical Association, the National Clinical Research Center for Skin and Immunological Diseases, and the Chinese Systemic Lupus Erythematosus Research Collaboration Group.^5^
- Disease duration ≤12 months.
- Patients in Group-B were diagnosed with autoimmune diseases in Affiliated Hospital of Hebei University.
- Complete and non-missing were available for both groups.

### Exclusion criteria:

- Complicated with infection, tumor, or drug-induced lupus.
- Pregnant or lactating women.
- Patients with end-stage renal disease, heart failure, or liver failure.
- Severe dysfunction of vital organs such as heart, liver, or kidneys.
- Received immunosuppressive therapy before admission.
- Venous blood(5 mL) was collected from the cubital vein of each patient upon admission. ANA titer, anti-ds-DNA, anti-nRNPSm, and Sm antibodies were detected using enzyme-linked immunosorbent assay (ELISA).

### Outcome measures:

The positivity of ANA, anti-ds-DNA, anti-nRNPSm, and Sm was defined as follows: ANA ≥1:80, anti-ds-DNA ≥50 IU/mL, anti-nRNPSm >20 U/mL, and Sm >20 U/mL. The positive and negative rates of the four indicators were compared between the two groups. The diagnostic value of individual indicators and their combined detection for early atypical SLE was analyzed.

### Statistical analysis:

Statistical analysis was performed using SPSS 27.0 software. Categorical data were presented as (*n*, %) and compared using the χ² test (for gender) or *Kappa* test (for antibody positive/negative rates). Continuous data were expressed as () and compared using independent samples *t*-test (for age). Logistic regression analysis was used to identify independent influencing factors. The diagnostic value of individual indicators and their combined detection was evaluated using receiver operating characteristic (ROC) curves, with the area under the curve (AUC) indicating predictive efficacy: 0.7–0.85 for moderate efficacy and >0.85 for good efficacy. A *P*-value <0.05 was considered statistically significant.

## RESULTS

*Kappa* test showed statistically significant differences in the positive rates of ANA, anti-ds-DNA, anti-nRNPSm, and Sm antibodies between the two groups (*P*<0.05). Details are shown in Table-I. Variables were coded prior to Logistic regression analysis, with patient Group-As the dependent variable and the four antibodies in Table-I as independent variables. Detailed coding is shown in Table-II.

**Table-I:** Comparison of ANA, Anti-ds-DNA, Anti-nRNPSm and Sm Test Results Between the Two Groups (n, %).

Group	n	ANA		ds-DNA		Anti-nRNPSm		Sm	
		Positive	Negative	Positive	Negative	Positive	Negative	Positive	Negative
Group-A	49	44 (89.8)	5 (10.20)	30 (61.22)	19 (38.78)	31 (63.27)	18 (36.73)	28 (57.14)	21 (42.86)
Group-B	66	26 (39.39)	40 (60.61)	28 (42.42)	38 (57.58)	22 (33.33)	44 (66.67)	22 (33.33)	44 (66.67)
Kappa Value	/	0.478		0.184		0.296		0.237	
P Value	/	<0.001		0.046		0.001		0.011	

**Table-II:** Variable Coding for Logistic Regression Analysis.

Factor	Variable Type	Coding
Group	Dependent Variable	1=Group-A, 2=Group-B
ANA	Independent Variable	1=Positive, 2=Negative
ds-DNA	Independent Variable	1=Positive, 2=Negative
Anti-nRNPSm	Independent Variable	1=Positive, 2=Negative
Sm	Independent Variable	1=Positive, 2=Negative

Logistic regression analysis showed that ANA, anti-ds-DNA, anti-nRNPSm, and Sm were independent influencing factors for early atypical SLE (*P*<0.05). Details are shown in Table-III. The AUC values of ANA, anti-ds-DNA, anti-nRNPSm, and Sm for diagnosing early atypical SLE were 0.752, 0.594, 0.650, and 0.619, respectively. Their diagnostic sensitivities and specificities were 89.8%/60.6%, 61.2%/57.6%, 63.3%/66.7%, and 57.1%/66.7%, with diagnostic accuracies and recall rates of 62.9%/89.8%, 42.6%/99.6%, 58.5%/63.3%, and 56.0%/57.1%, and diagnostic probabilities of 0.68, 0.50, 0.53, and 0.56. For combined detection, the four indicators were incorporated into a Logistic regression model and combined based on regression coefficients, ROC curve analysis showed that the combined diagnosis had an AUC of 0.856, a predictive sensitivity of 87.8%, a specificity of 71.2%, a predictive accuracy of 69.4%, a recall rate of 87.8%, and a predictive probability of 0.79. Details are shown in Table-IV and Fig.1, 2, and 3.

**Table-III:** Logistic Regression Analysis Results of ANA, Anti-ds-DNA, Anti-nRNPSm, and Sm.

Factor	B	Standard Error	Wald χ²	Degrees of Freedom	Significance	Exp(B)	95% CI for Exp(B)	
							Lower limit	Upper limit
ANA	1.317	0.287	21.014	1	<0.001	3.734	2.126	6.558
ds-DNA	0.511	0.248	4.261	1	0.039	1.667	1.026	2.707
Anti-nRNPSm	0.615	0.247	6.183	1	0.013	1.850	1.139	3.005
Sm	0.620	0.250	6.159	1	0.013	1.859	1.139	3.035

**Table-IV:** Diagnostic Value of ANA, Anti-ds-DNA, Anti-nRNPSm, Sm, and Their Combined Detection for Early Atypical SLE.

Test Variable	AUC	Standard Error	Asymptotic Significance	Asymptotic 95% CI		Sensitivity	Specificity	Youden Index	Precision	Recall	F1 Value
				Lower limit	Upper limit						
ANA	0.752	0.037	<0.001	0.679	0.825	89.8	60.6	0.504	62.9	89.8	0.74
ds-DNA	0.594	0.047	0.044	0.503	0.685	61.2	57.6	0.188	42.6	99.6	0.60
Anti-nRNPSm	0.650	0.045	0.001	0.561	0.739	63.3	66.7	0.300	58.5	63.3	0.61
Sm	0.619	0.046	0.010	0.529	0.710	57.1	66.7	0.238	56.0	57.1	0.57
Four-Indicator Combination	0.856	0.035	<0.001	0.787	0.925	87.8	71.2	0.590	69.4	87.8	0.78

**Fig.1:**
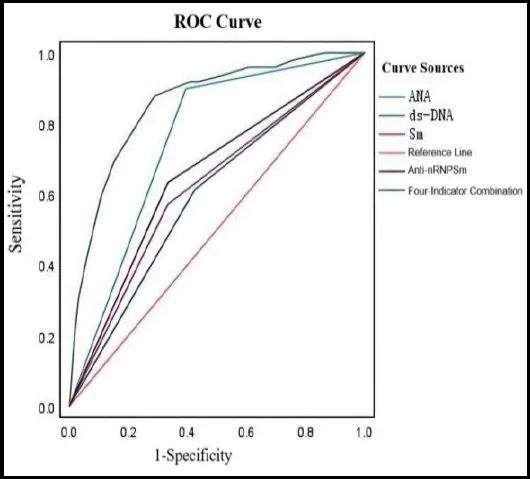
ROC Curves for the Diagnosis of Early Atypical SLE Using ANA, Anti-ds-DNA, Anti-nRNPSm, Sm, and their combined detection.

**Fig.2:**
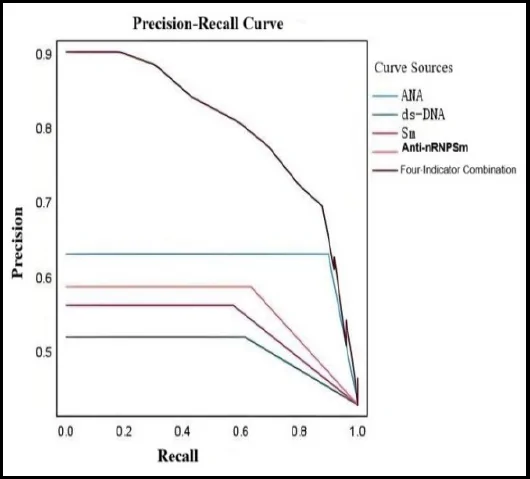
Precision-Recall Curves for the Diagnosis of Early Atypical SLE Using ANA, Anti-ds-DNA, Anti-nRNPSm, Sm, and their combined detection.

**Fig.3:**
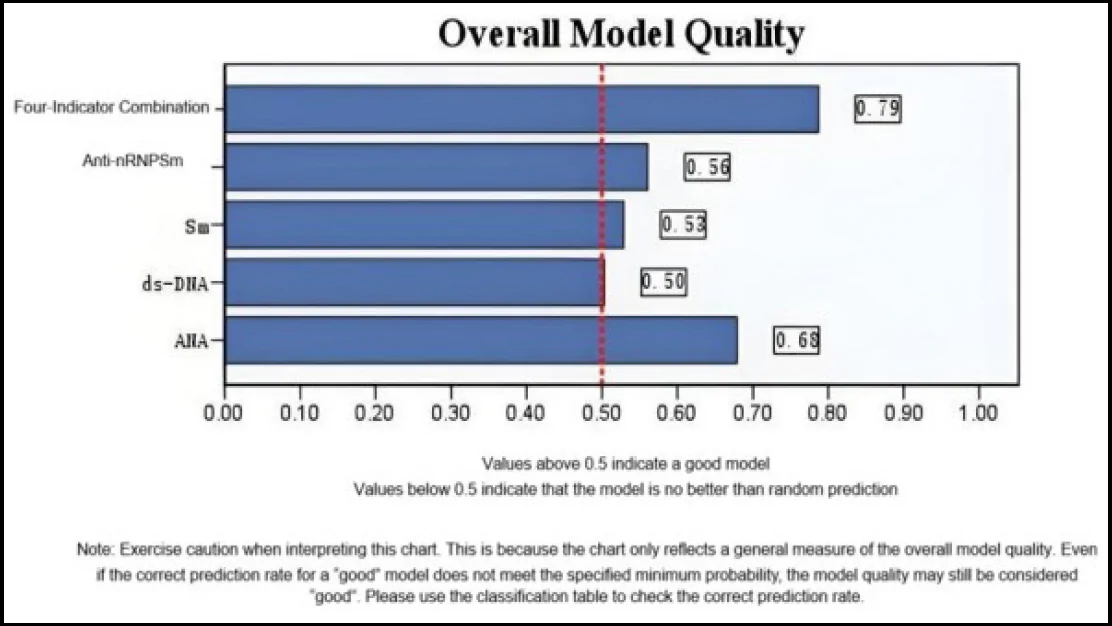
Bar Chart of Diagnostic Probabilities for ANA, Anti-ds-DNA, Anti-nRNPSm, Sm, and their combined detection.

Significant differences in AUC were observed between ANA and anti-ds-DNA, Sm, or the four-indicator combination (*P*<0.05); between anti-ds-DNA and the four-indicator combination (*P*<0.05); and between anti-nRNPSm, Sm and the four-indicator combination (*P*<0.05). Details are shown in Table-V.

**Table-V:** Comparison of AUC Differences Between ANA, Anti-ds-DNA, Anti-nRNPSm, Sm, and their combined detection.

AUC Result Comparison	Z Value	P Value
ANA vs. ds-DNA	2.496	0.013
ANA vs. Anti-nRNPSm	1.790	0.074
ANA vs. Sm	2.235	0.025
ANA vs. Four-Indicator Combination	-3.866	<0.001
ds-DNA vs. Anti-nRNPSm	-0.863	0.388
ds-DNA vs. Sm	-0.384	0.701
ds-DNA vs. Four-Indicator Combination	-4.772	<0.001
Anti-nRNPSm vs. Sm	0.440	0.660
Anti-nRNPSm vs. Four-Indicator Combination	-4.196	<0.001
Sm vs. Four-Indicator Combination	-5.230	<0.001

## DISCUSSION

This study found statistically significant differences in the positive rates of ANA, anti-ds-DNA, anti-nRNPSm, and Sm antibodies between patients with early atypical SLE (Group-A) and those with other autoimmune diseases (Group-B). Subsequent Logistic regression analysis confirmed that these four antibodies are independent risk factors for early atypical SLE.^6^,^7^ The core pathological mechanism of SLE involves an abnormal immune response of the body to self-nuclear antigens, which triggers immune complex deposition and inflammatory reactions. In the early atypical stage, although clinical symptoms are not yet fully manifested, abnormal activation of the immune system has already initiated, resulting in the specific expression of related autoantibodies.^8^,^9^ Littlejohn EA et al.^10^ reported that in the early stage of SLE, dendritic cells are abnormally activated and present self-nuclear antigens to T lymphocytes. Activated T cells further promote the proliferation and differentiation of B lymphocytes, which secrete a large number of ANA antibodies, resulting in a significant increase in ANA titer.

However, nuclear antigens may be exposed and induce immune responses in various autoimmune diseases. A study by Mora VP et al.^11^ demonstrated that in the early atypical stage of SLE, impaired clearance of apoptotic cells leads to the release of nucleosomes. As a core antigen within nucleosomes, ds-DNA induces B cells to produce specific anti-ds-DNA antibodies. At this early stage, the extent of impaired apoptotic cell clearance is mild, with a limited release of nucleosomes, leading to low expression levels of anti-ds-DNA antibodies.^12^,^13^ A study by Sasi S et al.^14^ showed that abnormal activation of immune cells induces structural changes in small nuclear ribonucleoprotein particles within the nucleus, exposing novel epitopes and eliciting specific immune responses that produce the corresponding antibodies. Anti-nRNPSm and Sm antibodies target small nuclear ribonucleoprotein particles, which participate in key physiological processes including RNA splicing.^15^,^16^ Similar to anti-ds-DNA antibodies, the extent of epitope exposure is limited in the early stage, resulting in low expression levels of these antibodies.^17^

For single-indicator diagnosis of early atypical SLE, the AUC values of ANA, anti-ds-DNA, anti-nRNPSm, and Sm antibodies were 0.752, 0.594, 0.650, and 0.619, respectively, with diagnostic probabilities of 0.68, 0.50, 0.53, and 0.56. The high sensitivity of ANA titer confers strong “screening value,” rendering it a preliminary screening marker for early atypical SLE that can effectively identify potential cases and reduce missed diagnoses. However, its low specificity means it cannot be used as a sole diagnostic criterion, as it is prone to misdiagnosis due to cross-reactivity with other autoimmune diseases. In contrast, the single-indicator diagnostic efficacy of anti-ds-DNA, anti-nRNPSm, and Sm antibodies is modest, with all AUC values below 0.7. Notably, the AUC of anti-ds-DNA antibody is close to 0.5, the threshold for non-diagnostic value, indicating that the clinical utility of these antibodies alone for diagnosing early atypical SLE is limited.

This finding is generally consistent with the conclusions of most studies both domestically and internationally.^18^ When these four antibodies were incorporated into a Logistic regression model and combined according to regression coefficients, ROC curve analysis demonstrated that the combined detection achieved an AUC of 0.856, with a predictive sensitivity of 87.8%, a specificity of 71.2%, and a predictive probability of 0.79—values significantly higher than those of individual antibodies. This finding clearly demonstrates that combined detection of the four antibodies can markedly enhance the diagnostic performance for early atypical SLE. The synergistic mechanism of combined detection may lie in the fact that the four antibodies target distinct immunological targets in the pathological process of SLE, thereby forming a complementary effect.

ANA primarily reflects the overall activation of the body’s immune response to nuclear antigens; anti-ds-DNA antibodies are closely associated with immune complex formation and renal injury in SLE; anti-nRNPSm antibodies participate in the dysregulation of immune responses in SLE; and Sm antibodies target the core components of nucleosomes. Their combined detection comprehensively covers the immunopathological features of early atypical SLE, mitigating the limitations of single-target detection.^19^,^20^ The Logistic regression model quantitatively allocates diagnostic weights to each antibody based on regression coefficients, enhancing the diagnostic contribution of high-value antibodies (e.g., ANA) while compensating for the insufficient sensitivity of low-performance individual antibodies (e.g., anti-ds-DNA), thus achieving a synergistic enhancement of diagnostic performance.

### Limitations:

This study has several limitations. Firstly, the relatively small sample size may constrain the statistical power of the findings, precluding in-depth subGroup-Analyses. Second, as a single-center retrospective study, all enrolled patients were from our hospital, potentially introducing selection bias. The external validity of the findings thus requires validation through multicenter prospective studies. Thirdly, the use of a single detection method (ELISA for all four antibodies) could compromise result accuracy. Given that different detection methods may exhibit differential diagnostic performance, no comparative analysis of alternative detection methods was conducted. Finally, this study did not investigate the dynamic changes in these antibodies. Their temporal changes during disease progression and prognostic predictive value in patients with early atypical SLE remain unclear.

## CONCLUSIONS

ANA, anti-ds-DNA, anti-nRNPSm, and Sm antibodies are independent influencing factors for early atypical SLE. Combined detection of these four antibodies may markedly enhance diagnostic performance.

### Recommendations:

Future studies should remedy these shortcomings and investigate future research avenues, offering more diverse insights for the management of early atypical SLE.

### Authors’ Contributions:

**JZ:** Designed the study and conceived the survey, are responsible and accountable for the accuracy and integrity of the work. **ML** and **QL:** data collection, analyzed clinical data. Critical review. **DC** and **MZ:** Literature search, Sorted the data, significantly revised this manuscript. All authors have read and approved the final manuscript.
